# Gutenberg Gait Database, a ground reaction force database of level overground walking in healthy individuals

**DOI:** 10.1038/s41597-021-01014-6

**Published:** 2021-09-02

**Authors:** Fabian Horst, Djordje Slijepcevic, Marvin Simak, Wolfgang I. Schöllhorn

**Affiliations:** 1 Department of Training and Movement Science, Institute of Sport Science, Johannes Gutenberg-University Mainz, Mainz, Germany; 2 Department of Media & Digital Technologies, Institute of Creative Media Technologies, St. Pölten University of Applied Sciences, St. Pölten, Austria

**Keywords:** Scientific data, Machine learning, Biomedical engineering, Outcomes research

## Abstract

The Gutenberg Gait Database comprises data of 350 healthy individuals recorded in our laboratory over the past seven years. The database contains ground reaction force (GRF) and center of pressure (COP) data of two consecutive steps measured - by two force plates embedded in the ground - during level overground walking at self-selected walking speed. The database includes participants of varying ages, from 11 to 64 years. For each participant, up to eight gait analysis sessions were recorded, with each session comprising at least eight gait trials. The database provides unprocessed (raw) and processed (ready-to-use) data, including three-dimensional GRF and two-dimensional COP signals during the stance phase. These data records offer new possibilities for future studies on human gait, e.g., the application as a reference set for the analysis of pathological gait patterns, or for automatic classification using machine learning. In the future, the database will be expanded continuously to obtain an even larger and well-balanced database with respect to age, sex, and other gait-specific factors.

## Abstract (summary)

**Table:** 

Measurement(s)	ground reaction force • centre of pressure • walking behavior • gait measurement • Normal Gait
Technology Type(s)	force plate • Sensor Device
Factor Type(s)	age • sex • walking speed
Sample Characteristic - Organism	Homo sapiens
Sample Characteristic - Environment	laboratory environment

Machine-accessible metadata file describing the reported data: 10.6084/m9.figshare.15112488

## Background & Summary

The ability to walk is crucial for human mobility and is closely related to quality of life independent of age and sex^1–4^. The fear of losing the ability to walk is often considered as the most important concern of people after an accident or diagnosis, such as stroke^5^ or Parkinson’s disease^6,7^, and emphasizes the importance of walking for self-determined everyday life. In the healthcare sector, great efforts are made to prevent, diagnose, and rehabilitate limitations or even loss of independence due to gait impairments^1,3,8^. Three-dimensional instrumented gait analysis (3DGA) using video- or infrared-based motion capture systems and force plates is frequently used to objectively and quantitatively describe human locomotion. Consequently, 3DGA supports clinicians, therapists, and researchers in the standardized assessment of gait deviations and the detection of changes caused by orthopedic or physiotherapeutic interventions^9,10^. An evaluation using instrumented gait analysis is frequently accompanied by a large amount of data^8,11,12^, which are difficult to comprehend due to their multi-dimensional and multi-correlated nature^13–15^. The interpretation of such data can be a challenge even for experienced clinicians. Therefore, different approaches have been developed in recent years to facilitate the generation of meaningful clinical conclusions from 3DGA data and to support decision-making of clinical experts. Such approaches are based on, e.g., gait indexes^15^, multivariate statistical analysis^13^, and machine learning (ML)^8,11,12,16^. The latter are able to take into account and combine several time-continuous gait variables at once. These approaches can also support more experienced clinicians, whose evaluations are often based on subjective experiences with specific patient groups, by providing an objective perspective on the data.

In recent years, several ML-based approaches have been published that can assist clinicians in identifying individual gait characteristics^17,18^ and classifying specific gait patterns into clinically relevant categories^16,19^, e.g., stroke^20^, Parkinson’s disease^21^, cerebral palsy^22^, or specific functional gait disorders^23^. Although previous ML-based approaches provided promising results with respect to classification accuracy, these models have so far often been trained and evaluated on relatively small and well-controlled datasets as well as applied to simple classification tasks (e.g., healthy controls vs. Parkinson’s disease). The question of whether it is possible to train ML models that meet clinical requirements in terms of robustness, transparency, and generalizability has rarely been investigated. This has so far hindered broader clinical application and acceptance of ML models. The availability of sufficient and high-quality data is an important prerequisite for the training of reliable ML models. However, the availability of 3DGA data is often a limitation in practice. Among other authors^24,25^, we also made 3DGA data available to the public^26–30^ in previous studies^17,31–34^. However, different data processing procedures and data structures were used in these studies, making collaborative use of the data difficult. In recent years, a rather small number of annotated large-scale datasets have been made publicly accessible^35^. Publicly available datasets can be used to train more robust models. In practice, even with a large-scale dataset, such as GaitRec^35^, data from individuals without gait pathology (healthy controls) represent a bottleneck. One reason for the scarcity of such data is that most gait analysis laboratories are located at clinics and usually record and examine only patients with pathological gait patterns.

In order to address this shortcoming, we provide - with the Gutenberg Gait Database - the gait data from healthy controls collected in our laboratory over the past seven years. The data is provided in a uniform format to allow for a continuously growing and publicly accessible database. The overall goal is to bridge existing gaps in publicly available gait datasets. Thereby, we aim at creating a basis for reliable ML models that can be used as decision-support system in clinical practice and research. Based on this goal, we prepared the processed data in such a way that it can be merged and used in conjunction with the GaitRec dataset^35^. In addition, the size and quality of the database allow it to serve as an extension of the study population in gait-related research areas, e.g., shoe and insole research^36^, security systems based on biometric recognition^37^, gait-based fatigue^38^ and emotion^39^ detection in psychological and sport-related contexts. In this setting our database can be used in various ways, e.g., as reference data or as source for automatic outlier detection. From a more epistemological point of view, the continuously growing database will also allow increasing flexibility in dealing with much more diverse questions related to human gait. Questions concerning population-motivated research^40^, problems of specific groups^41^, or the complexity of individual case-oriented time series^31,32^ will be put on a broader data-based foundation over time.

The Gutenberg Gait Database provides exclusively force plate data, namely ground reaction force (GRF) and center of pressure (COP) signals. The current best practice in clinical gait analysis describes a patient’s gait using a combination of force plate data with kinematic and electromyographic data. However, kinematic and electromyographic data are prone to several difficulties, such as inconsistencies due to differences in anthropometric characteristics of participants, experience of investigators, measurement protocols, and laboratory settings^42–44^. This makes it more difficult to create a homogeneous, large-scale, and high-quality dataset compared to using less interference-prone data, such as GRF signals^45,46^. Therefore, the use of force plate data offers advantages for the development of ML models for gait analysis, although the provided information appears to be reduced in comparison to kinematic data. However, previous studies^23,47^ investigating ML methods for automated classification of gait impairments based on force plate data showed promising results suggesting their suitability for clinical applications.

## Methods

### Datasets

**Fig. 1:**
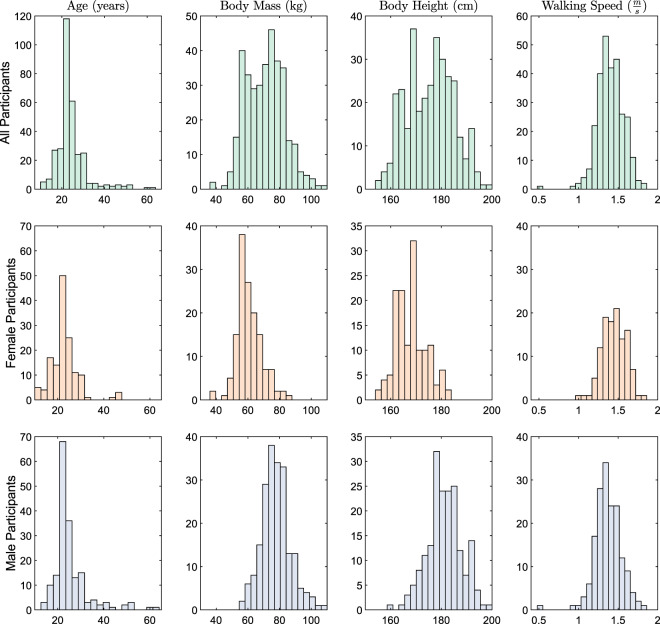
Frequency distribution of age, body mass, body height, and walking speed for all (upper panel), female (middle panel), and male (lower panel) participants. The distributions are based on the values of the initial session of each participant. For the waking speed, the mean values of the gait trials of the initial session are shown.

The Gutenberg Gait Database combines datasets from five already published studies on human gait^17,31–34^ and data from five unpublished studies. A total sample of 350 participants (142 female, 205 male, and 3 unknown) aged between 11 and 64 years is included. Prior to the recording, all participants reported that they did not have any gait pathology and were not suffering from any injuries or diseases that affected gait. Table 1 summarizes demographic details for each individual dataset and the total database. Figure 1 shows the overall and sex-specific distributions of age, body mass, body height, and walking speed for the database.

**Table 1:** Demographic details of individual datasets and the total database.

Dataset	ID	N	Sex (male/female)	Age (years) Mean (SD)	Body Mass (kg) Mean (SD)	Body Height (m) Mean (SD)
Horst *et al*. (2016)^31^	1	8	2/6	23.3 (2.4)	65.9 (8.0)	1.73 (0.07)
Horst *et al*. (2017)^33^	2	9	6/3	27.4 (3.0)	73.2 (13.3)	1.74 (0.11)
Horst *et al*. (2017)^32^	3	128	76/52	23.8 (9.0)	71.3 (13.0)	1.77 (0.08)
Horst *et al*. (2019)^17^	4	57	28/29	23.1 (2.7)	67.9 (11.3)	1.74 (0.10)
Burdack *et al*. (2020)^34^*	5	33	14/19	25.1 (6.7)	65.1 (9.6)	1.71 (0.09)
Unpublished Study 1	6	38	38/0	28.0 (10.8)	78.2 (9.7)	1.81 (0.04)
Unpublished Study 2	7	26	26/0	24.7 (2.9)	79.8 (8.8)	1.82 (0.07)
Unpublished Study 3	8	25	0/25	23.3 (4.2)	62.6 (7.6)	1.67 (0.05)
Unpublished Study 4	9	23	15/8	24.0 (2.5)	69.1 (10.5)	1.77 (0.10)
Unpublished Study 5	10	3	—	—	72.4 (7.8)	—
**Total**	**10**	**350**	**205/142**	**24.2 (7.0)**	**70.7 (12.0)**	**1.76 (0.09)**

*For dataset 2 and dataset 5 the experimental protocol was identical. In the analysis conducted by

Burdack *et al*. (2020)^34^, the data from both datasets were analysed together.

All studies (published and unpublished) were carried out according to the Declaration of Helsinki at the Johannes Gutenberg-University in Mainz (Germany). All participants were informed about the experimental protocol and provided their written informed consent to participate in the study. The approval from the ethical committee of the medical association Rhineland-Palatinate in Mainz (Germany) was received.

### Data recording & Experimental protocol

Bi-lateral analog force plate signals were recorded by asking participants to walk at their preferred (self-selected) walking speed on a level and approximately 10 m long walkway. Two force plate configurations were used: (i) an inline configuration using two centrally embedded force plates (Kistler, Type 9287CA, Switzerland) and (ii) a staggered configuration using two force plates (Kistler, Type 9286AA, Switzerland) integrated in a wooden walkway.

For both force plate configurations, the analog force plate signals were amplified (Kistler, Type 5233 A, Switzerland) and converted to digital signals using a sampling frequency of 1,000 Hz. A data acquisition system (Kistler, Type 5695, Switzerland) with a 16-bit analog-digital converter (Measurement Computing Corporation, Type USB-2533, USA) was used with a signal input range of ±10 V. Depending on the underlying experimental protocol, the walking speed was either estimated using (i) two light barriers with two photoelectric sensors (Imhof Timing, Germany) at a sampling frequency of 1,000 Hz or (ii) the three-dimensional pelvis marker trajectories captured by nine infrared cameras (Qualisys AB, Type Oqus 310, Sweden) at a sampling frequency of 250 Hz.

Participants were asked to perform gait trials to familiarize with the experimental setup and to determine an individual starting position for the gait analysis session. The number of familiarization trials differed between the experimental protocols. The exact number is specified for each study in Table 2. This procedure has already been shown to minimize the impact of targeting the force plates on the observed gait variables^48,49^. In addition, the participants were instructed to look at a symbol (neutral smiley) on the opposing wall of the laboratory to direct their attention away from the force plates and ensure a natural walk with an upright body position.

**Table 2:** Data recording and experimental protocol details of the individual datasets.

Dataset	ID	Force Plate Configuration	Walking Speed Estimation Method	Gait Analysis Sessions	Familiarization Trials	Gait Trials per Session	Total Number of Gait Trials
Horst *et al*. (2016)^31^	1	inline	infrared cameras	8	20(4)**	15	949
Horst *et al*. (2017)^33^	2	inline	infrared cameras	6	20(5)**	15	806
Horst *et al*. (2017)^32^	3	staggered	light barriers	1(2)*	5	10	1,737
Horst *et al*. (2019)^17^	4	inline	infrared cameras	1	20	20	1,130
Burdack *et al*. (2020)^34^	5	inline	infrared cameras	6	20(5)**	15	2,959
Unpublished Study 1	6	inline	—	1	10	10	377
Unpublished Study 2	7	staggered	light barriers	1	5	8	233
Unpublished Study 3	8	inline	—	1	10	15	374
Unpublished Study 4	9	inline	infrared cameras	1	5	10	231
Unpublished Study 5	10	inline	—	1	5	8	23
**Total**	**10**	**mixed**	**mixed**	**1–8**	**5–20**	**8–20**	**8,819**

*Forty-seven out of one hundred and twenty-eight participants attended a second gait analysis session.

**Numbers in parentheses () represent the number of familiarization trials performed by participants before follow-up sessions in experimental protocols with repeated gait analysis sessions.

During one gait analysis session, participants walked until a predefined number of valid gait trials were available. These gait trials were defined as valid by the assessor if the participant walked “naturally” (e.g., with respect to force plate targeting) and both force plates were hit cleanly. The predefined number of gait trials per session varied between the experimental protocols and ranged from 8 to 20 gait trials. The exact number for each experimental protocol is specified in Table 2. Depending on the experimental study design, one to eight gait analysis sessions were recorded per participant.

### Data processing

The three-dimensional GRFs (vertical, anterior-posterior, and medio-lateral) and the two-dimensional COPs (anterior-posterior and medio-lateral) were calculated based on the analog force plate signals. The database provides unprocessed (raw) and processed (ready-to-use) GRF and COP signals during the stance phase. The data processing procedure was coordinated with Horsak *et al*.^35^ so that the processing of the data in the Gutenberg Gait Database is identical to the GaitRec dataset. Thereby, we were able to prevent the obstacles that often exist in practice when using different datasets jointly. The main benefit for the community is the combined use of both data sources. We have, thus, eliminated a major disadvantage of the GaitRec dataset, namely ensuring that the number of healthy control participants is no longer a bottleneck.

For both settings, i.e., unprocessed and processed data, following pre-processing steps were performed. The offset of each analog force plate signal was corrected using the mean value of the first ten frames. The analog force plate signals were down-sampled to 250 Hz. The orientation of the medio-lateral and anterior-posterior GRF and COP signals were unified. Thus, medial and anterior forces were transformed to positive and lateral and posterior to negative values.

For the unprocessed (raw) data, we determined the signals in the following way. The stance phase was determined using a vertical GRF threshold of 25 N. The cropped GRF signals of the stance phase were used to calculate the COP signals.

For the processed (ready-to-use) data, we filtered the GRF signals using a second-order Butterworth bidirectional low-pass filter at a cut-off frequency of 20 Hz. The stance phase was determined based on the filtered GRF signals using a vertical GRF threshold of 25 N. For the processed COP signals, we filtered the unprocessed (raw) COP signals as well with a second-order Butterworth bidirectional low-pass filter at a cut-off frequency of 20 Hz. Furthermore, we cropped the filtered COP signals with a vertical GRF threshold of 80 N to avoid artifacts in COP calculation at small GRF signal values. In addition, the medio-lateral COP signals were mean-centered and anterior-posterior COP signals zero-centered. Each GRF and COP signal was time-normalized to 101 data points, corresponding to 100% stance phase. The GRF signals were normalized to the body weight, measured before each gait analysis session. The whole data processing was performed within the Matlab 2019a (The MathWorks, Inc., Natick, Massachusetts, USA) framework.

## Data Records

All published data are fully anonymized and are available online from figshare^50^. As already pointed out, we decided to follow the data processing procedure and data structure as well as the naming of the files according to the GaitRec dataset^35^. The data records consist of twenty files containing the GRF data for each gait trial (see Table 3) and one file containing the measured walking speed for each gait trial. In addition, we provide one file containing metadata for each gait analysis session, including additional participants’ information, e.g., class label, sex, age, body mass. All files are available as comma-separated value files (.csv). The twenty GRF data files are organized according to the following naming convention: “*GRF-type-processing-side*.csv”. The *type* denotes, whether the file holds the vertical (“F_V”), anterior-posterior (“F_AP”), medio-lateral (“F_ML”) or the anterior-posterior or medio-lateral COP (“COP_AP”, “COP_ML”) time-series. *Processing* denotes, if the files hold the unprocessed (raw) data (“RAW”) or the processed (ready-to-use) data (“PRO”). The *side* denotes, if the data are from the “left” or “right” body side. The common prefix for all files is “GRF-”. An example filename is: “GRF_F_V_RAW_left.csv”.

**Table 3:** Description of the data stored in the “GRF_*.csv” files. “*” for the associated file name is a placeholder for “right” and “left” (adapted from Horsak *et al*.^35^).

Variables	Associated file	Format	Dimension	Unit	Description
Vertical GRF	GRF_F_V-RAW_*.csv	double	1 × n	Newton	Unprocessed vertical ground reaction force
Anterior-posterior GRF	GRF_F_AP-RAW_*.csv	double	1 × n	Newton	Unprocessed breaking and propulsive shear force
Medio-lateral GRF	GRF_F_ML_RAW_*.csv	double	1 × n	Newton	Unprocessed medio-lateral shear force
COP anterior-posterior	GRF_COP_AP_RAW_*.csv	double	1 × n	Meter	Unprocessed COP coordinate in walking direction
COP medio-lateral	GRF_COP_ML_RAW_*.csv	double	1 × n	Meter	Unprocessed COP coordinate in medio-lateral direction
Vertical GRF	GRF-F_V_PRO_*.csv	double	1 × n	Multiple of body weight	Processed vertical ground reaction force
Anterior-posterior GRF	GRF_F_AP_PRO_*.csv	double	1 × n	Multiple of body weight	Processed breaking and propulsive shear force
Medio-lateral GRF	GRF-F_ML_PRO_*.csv	double	1 × n	Multiple of body weight	Processed medio-lateral shear force
COP anterior-posterior	GRF_COP_AP_PRO_*.csv	double	1 × n	Meter	Processed COP coordinate in walking direction
COP medio-lateral	GRF_COP_ML_PRO_*.csv	double	1 × n	Meter	Processed COP coordinate in medio-lateral direction
Walking Speed	GRF_walking_speed.csv	double	1 × n	$\frac{m}{s}$	Measured walking speed

n is either the number of frames during one step across the force plate for the unprocessed data (“RAW”) or a time-normalized vector of 101 points for the

processed (“PRO”) data. Note that the first four columns of each file hold the *DATASET_ID, SUBJECT_ID, SESSION_ID, and TRIAL_ID.*

Each of the “*GRF-type-processing-side*.csv” files is structured as a matrix with *T* rows × *K* columns (*T* = 8,819; *K* = 105 for “PRO” and *K* = 216 for “RAW”). Each row holds the data of one gait trial. The first column identifies each dataset (“DATASET_ID”), the second column each participant (“SUBJECT_ID”), the third column each gait analysis session (“SESSION_ID”), and the fourth column each single gait trial within a session (“TRIAL_ID”). The remaining columns contain the values of the GRF signals for each gait trial. Note that due to the non-normalized nature of the data and the resulting different time-series lengths in the “RAW” files, non-available numbers have been replaced by “NaN” to maintain a constant matrix-dimension.

The file holding the measured walking speed for each gait trial is named “GRF_walking_speed.csv”. The file is structured as a matrix with *T* rows × *L* columns (*T* = 8,819; *L* = 5). Each row holds the data of one gait trial. The first column identifies each dataset (“DATASET_ID”), the second column each participant (“SUBJECT_ID”), the third column each gait analysis session (“SESSION_ID”), and the fourth column each single gait trial within a session (“TRIAL_ID”). The fifth column contains the measured walking speed for each gait trial (“WALKING_SPEED”). The walking speed was not measured in datasets 6, 8, and 10. Non-available numbers have been replaced by “NaN” to maintain a constant matrix-dimension.

The metadata file, which contains additional participant and session-related information is named “GRF_metadata.csv” (see Table 4). The file is structured as a matrix with *S* rows × *M* columns (*S* = 661; *M* = 21). Here, the first three columns hold the DATASET_ID, SUBJECT_ID, and SESSION_ID, the other columns hold information such as sex, body mass, and age (see Table 4 for more details). Non-available numbers have been replaced by “NaN” to maintain a constant matrix-dimension.

**Table 4:** Description of the information stored in the metadata file (adapted from Horsak *et al*.^35^).

Categories/Variables	Format	Unit	Description
**Identifiers**			
DATASET_ID	integer	—	Unique identifier of a dataset
SUBJECT_ID	integer	—	Unique identifier of a participant
SESSION_ID	integer	—	Unique identifier of a gait analysis session
**Labels**			
CLASS_LABEL*	string	—	Annotated class labels
CLASS_LABEL_DETAILED*	string	—	Annotated class labels for subclasses
**Participant Metadata**			
SEX	binary	—	female = 0, male = 1
AGE	integer	years	Age at recording date
HEIGHT	integer	centimeter	Body height in centimeters
BODY_WEIGHT	double	$\frac{kg\;m}{{s}^{2}}$	Body weight in Newton
BODY_MASS	double	kg	Body mass
SHOE_SIZE	double	EU	Shoe size in the Continental European System
AFFECTED_SIDE*	integer	—	left = 0, right = 1, both = 2, none = NaN
**Trial Metadata**			
SHOD_CONDITION*	integer	—	barefoot & socks = 0, normal shoe = 1, orthopedic shoe = 2
ORTHOPEDIC_INSOLE*	binary	—	without insole = 0, with insole = 1
SPEED*	integer	—	slow = 1, self-selected = 2, fast = 3 walking speed class
READMISSION*	integer	—	indicates the number of readmission = 0 L n
SESSION_TYPE*	integer	—	initial = 1, control = 2, initial after readmission = 3
SESSION_DATE	string	—	date of gait analysis session in the format “DD-MM-YYYY hh:mm”
**Train-Test Split Information**			
TRAIN*	binary	—	is part ( = 1) or is not part ( = 0) of TRAIN
TRAIN_BALANCED*	binary	—	is part ( = 1) or is not part ( = 0) of TRAIN_BALANCED*
TEST*	binary	—	is part ( = 1) or is not part ( = 0) of TEST

*The metadata items highlighted by an asterisk were included primarily to ensure a consistent data structure between

the Gutenberg Gait Database and the GaitRec dataset^35^.

## Technical Validation

The force plates and the measurement equipment were calibrated by the manufacturer (Kistler, Switzerland) and regularly checked and serviced during laboratory practice. No specific procedure (e.g., such as the CalTester method) was used.

In addition, on each day when measurements were conducted, the proper functioning of the force plates and measuring equipment was ensured by the following procedure: (i) A 30 s recording without load on the force plates was taken and ensured that the signal noise was below ±1 N. (ii) The assessor performed a weight measurement to verify the proper amplification of the analog channels. (iii) The assessor walked along the 10 m analysis walkway with one foot contact on each force plate and verified that the GRF signals showed the characteristic curves.

**Fig. 2:**
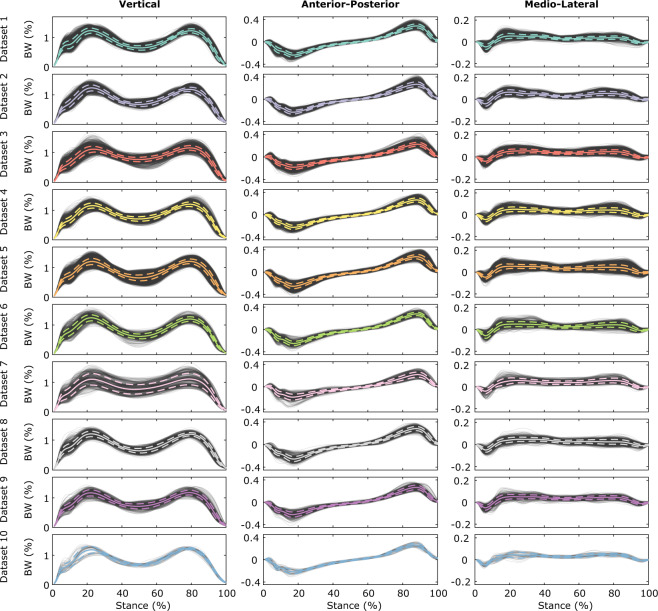
Visualization of vertical (left panel), anterior-posterior (central panel), and medio-lateral (right panel) force components of the body weight (BW)-normalized GRF measurements per dataset. Mean and standard deviation signals (calculated per dataset) are highlighted as solid and dashed colored lines.

**Fig. 3:**
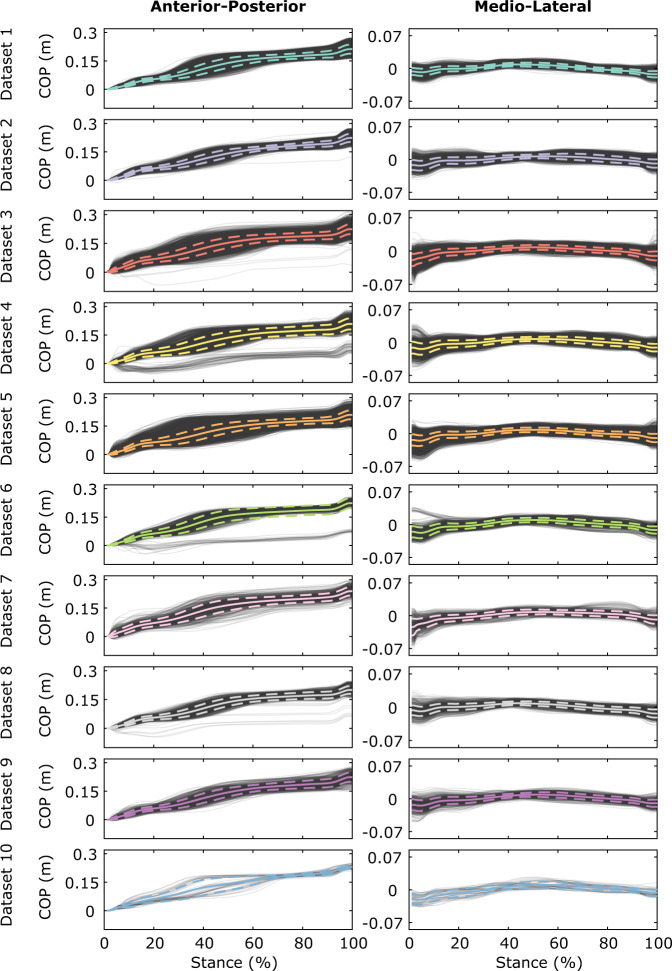
Visualization of zero-centered anterior-posterior (left panel) and mean-centered medio-lateral (right panel) components of the COP measurements per dataset. Mean and standard deviation signals (calculated per dataset) are highlighted as solid and dashed colored lines. We carefully inspected the gait trials where the signals differed considerably and made sure that these differences were not the result of measurement or calculation errors. Using the kinematic data, we were able to verify that the deviating signals were from gait trials of forefoot or midfoot walking participants.

For an impression of data integrity, the processed data for each dataset is shown in Fig. 2 (GRF) and Fig. 3 (COP).

## Usage Notes

The data are stored in *.csv files and can be easily imported into any software framework for further data analysis. We provide two scripts that allow a straightforward data import for Matlab (The MathWorks, Inc., Natick, Massachusetts, United States, 2019a) and Python (Python Software Foundation, 3.7). Additionally, two scripts (for Matlab and Python) are available for merging the GaitRec dataset^35^ and the Gutenberg Gait Database. For the GaitRec dataset the DATASET_ID is set to 0. Since the metadata files and the data files have the same structure, a simple consolidation can be achieved. The GaitRec dataset has a bottleneck in terms of healthy control participants. Merging the two datasets can compensate for this limitation and allow the data to be much more useful for future research. Merging the two data sources would increase the number of healthy controls from 211 to 561, which approximately corresponds to the cardinality of the gait disorder classes: hip (N = 450), knee (N = 625), ankle (N = 627), calcaneus (N = 382).

## Data Availability

A custom script for tracing and replicating the used processing of the force plate data in Matlab (The MathWorks, Inc., Natick, Massachusetts, United States, 2019a) and custom scripts for importing and merging (with the GaitRec dataset) the data in Matlab (The MathWorks, Inc., Natick, Massachusetts, United States, 2019a) and Python (Python Software Foundation, 3.7) are publicly available at figshare^50^.
